# Pipeline embolization devices for the treatment of nonsaccular aneurysms in pediatric patients

**DOI:** 10.3389/fneur.2023.1115618

**Published:** 2023-02-28

**Authors:** Jintao Han, Fei Liang, Yuxiang Zhang, Yupeng Zhang, Shikai Liang, Haoyu Zhu, Yuzhou Chang, Chao Ma, Lian Liu, Zichang Jia, Chuhan Jiang

**Affiliations:** ^1^Department of Interventional Radiology and Vascular Surgery, Peking University Third Hospital, Beijing, China; ^2^Department of Neurosurgery, Beijing Neurosurgical Institute and Beijing Tiantan Hospital, Capital Medical University, Beijing, China; ^3^Department of Neurosurgery, Beijing Tsinghua Changgung Hospital, School of Clinical Medicine, Tsinghua University, Beijing, China

**Keywords:** pediatric diseases, pipeline embolization device, nonsaccular aneurysms, neurovascular diseases, endovascular treatment

## Abstract

**Objective:**

Pediatric nonsaccular aneurysms are rare but challenging lesions; pipeline embolization devices (PEDs) are their potential treatment option. In this study, we aimed to evaluate the safety and efficacy of PEDs for treatment of these aneurysms.

**Methods:**

We retrospectively selected pediatric patients with nonsaccular aneurysms treated using PEDs between June 2015 and July 2021 from our prospectively maintained database. For each patient, demographics, aneurysm characteristics, procedure details, and clinical and angiographic follow-up data were collected and summarized.

**Results:**

This study included 16 pediatric patients with 16 nonsaccular aneurysms treated with PEDs. A median clinical follow-up time of 1,376 days was achieved in 93.75% of the patients. The complication rate of the included patients was 25%, with two patients developing mass effect, one patient undergoing major ischemic stroke, and one patient experiencing stent foreshortening after the procedure. The complete occlusion rate of aneurysms without any neurologic sequelae was 93.33%, with a median angiographic follow-up period of 246 days. The mortality rate was 6.25%.

**Conclusions:**

The use of PEDs to treat pediatric nonsaccular aneurysms is feasible, with a high rate of complete occlusion of the aneurysm and favorable follow-up outcomes.

## Introduction

Pediatric nonsaccular intracranial aneurysms can cause symptoms, such as headaches, mass effect, focal neurologic deficits, and seizures, and can even cause subarachnoid hemorrhage or stroke; in severe cases, this can endanger the child's life (1, 2). Although this disease is rare, it can be challenging for both neurosurgeons and neurointerventionists. These lesions generally have a dissecting or fusiform morphology, a large or giant size, and rapid disease progression (2, 3). With the advancement of interventional techniques and imaging technology, endovascular therapy has become one of the main methods for treating aneurysms (4–7). The pipeline embolization device (PED; Medtronic Neurovascular; Irvine, California, USA) is a flow diverter used for the endovascular treatment of intracranial aneurysms. Previous case reports and case series have shown that PEDs may provide a paradigm shift in the treatment of intracanal aneurysms in pediatric patients (8–10). However, these studies did not individually describe the treatment outcomes of nonsaccular aneurysm patients treated with PEDs.

In this study, we retrospectively collected patient demographics, aneurysm characteristics, procedural details, and clinical and angiographic outcomes of pediatric patients with nonsaccular aneurysms treated with PEDs, to evaluate the safety and efficacy of PEDs for their management.

## Materials and methods

### Patient selection

We retrospectively selected patients admitted between June 2015 and July 2021 from our prospectively maintained database. The inclusion criteria were as follows: (1) age ≤ 18 years at the time of admission, (2) intracranial aneurysms successfully treated with PEDs, and (3) nonsaccular aneurysms. The need for institutional review board approval was waived, considering the retrospective nature of the study and the use of de-identified data.

### Endovascular treatment

All pediatric patients were thoroughly evaluated pre-operatively by two or more experienced neurointerventionists for PED treatment. Patients were administered aspirin (100 mg/day) and clopidogrel (75 mg/day) orally if older than 5 years or aspirin (50 mg/day) and clopidogrel (37.5 mg/day) orally if aged 4 years. The patients were premedicated 7 days before the procedure. Thromboelastography was used to examine the inhibition rate of platelet activity before surgery.

Generally, Marksman or Phenom 27 microcatheters (ev3; Irvine, California, USA) were used for PED implantation, and pre-jailed Echelon-10 microcatheters (ev3; Irvine, California, USA) were used for adjunctive coiling with triaxial guide-catheter systems. For vertebrobasilar junction aneurysms, PEDs were implanted in the dominant vertebral artery and were used to occlude the non-dominant vertebral artery to reduce the risk of post-operative aneurysm rupture and to increase the embolization rate.

The operations were performed under general anesthesia and systemic heparinization. After the operation, the patients were administered continuous aspirin and clopidogrel orally for 6 months. Aspirin was orally administered until the aneurysms were completely embolized. The patients were followed up at 6, 12, and 18 months after the procedure.

### Acquisition of clinical and angiographic variables

Patient demographics, aneurysm characteristics, procedural details and follow-up outcomes were obtained from medical records, angiographic imaging and telephone questionnaires. Major complications were defined as those that lasted for more than 7 days. The O'Kelly-Marotta grading scale was used to assess angiographic outcomes (11). Patient physical condition was evaluated using the modified Rankin Scale (mRS): an mRS score of 0–2 was considered a favorable outcome.

### Statistical analysis

For categorical variables, data are presented as absolute values followed by percentages. For continuous variables, data are shown as mean ± standard deviation or median with interquartile range (IQR). Statistical analyses were performed using R 4.0.4 (R Foundation for Statistical Computing; Vienna, Austria). Statistical significance was set at *P* < 0.05.

## Results

### Demographics and aneurysm characteristics

We retrospectively collected data from 16 patients, each with 1 nonsaccular aneurysm treated with PEDs. The majority of patients were male, with a mean age of 13 years. Symptomatic aneurysms account for 75% of all the aneurysms. The mean maximum diameter of the included aneurysms was 25.21 ± 8.84 mm. Ten aneurysms were located in posterior circulation. Two aneurysms recurred. The baseline mRS was 0–2. Patient demographics and aneurysm characteristics are summarized in Table 1.

**Table 1:** Patient demographics and aneurysm characteristics.

**Variables**	**Values**
Patients (*n*)	16
Age (years), mean ± SD	13 ± 4
Sex (females)	4 (25%)
**Clinical presentation**	
Incidental	4 (25%)
Headache	8 (50%)
Epilepsy	1 (6.25%)
Diplopia	1 (6.25%)
Dizziness	1 (6.25%)
Numbness in the left limb	1 (6.25%)
Aneurysm maximum diameter (mm), mean ± SD	25.21 ± 8.84
**Aneurysm location**	
C4 segment of ICA	2 (12.5%)
C6 segment of ICA	2 (12.5%)
C7 segment of ICA	1 (6.25%)
M1 segment of MCA	1 (6.25%)
V4 segment of VA	2 (12.5%)
Vertebrobasilar junction	3 (18.75%)
Basilar trunk	3 (18.75%)
P2 segment of PCA	2 (12.5%)
Recurrent aneurysms	2 (12.5%)
**Baseline mRS**	
mRS 0-2	16 (100%)

SD, standard deviation; ICA, internal carotid artery; MCA, middle cerebral artery; VA, vertebral artery; PCA, posterior cerebral artery; mRS, modified Rankin Scale.

### Procedural details and follow-up outcomes

Twenty-eight PEDs were used to treat these aneurysms, including two aneurysms requiring three overlapping PEDs and one aneurysm requiring four overlapping PEDs. Seven aneurysms were treated using PEDs and adjunctive coils. The median clinical follow-up time was 1,376 days (IQR, 1,136–1,710) in 16 patients. The complication rate in the included patients was 25%. In addition, 93.75% of the patients achieved favorable outcomes.

Two patients developed a mass effect after surgery. One of the patients was a 12-year-old male patient with a maximum aneurysm diameter of 35.5 mm located at the basilar trunk artery. The patient was treated with 4 overlapping PEDs without adjunctive coils. The PEDs were implanted successfully, but the patient died of respiratory and circulatory failure caused by mass effect 3 days after the procedure. Another patient having mass effect complication is described in Section Case presentation 1. One patient experienced major ischemic stroke after the procedure. A 10-year-old male patient had a vertebrobasilar junction aneurysm with a maximum diameter of 27.3 mm. Overlapping of two PEDs with adjunctive coils was performed successfully to treat the aneurysm. Two months after the operation, the patient's left limb muscle strength decreased to grade III from grade V and he suffered left facial paralysis. The patient recovered 9 days after conservative treatment and the mRS score was 0 at the latest follow-up. One patient experienced stent foreshortening post-operatively. A 17-year-old male patient had a basilar trunk aneurysm with a maximum diameter of 27.5 mm, and one PED without adjunctive coils was used for its management. At 18 months post-operatively, stent foreshortening was found, and another PED was overlapped to retreat the aneurysm.

The median angiographic follow-up period was 246 days (IQR, 171–459) in 15 patients. The complete occlusion rate of aneurysms without neurological deficits was 93.33%. The procedural details and follow-up outcomes are summarized in Table 2.

**Table 2:** Procedural details and follow-up outcomes.

**Parameters**	**Values**
**PED number**	
1	8 (50%)
2	5 (31.25%)
≥3	3 (18.75%)
Adjunctive coils	7 (43.75%)
Clinical follow-up time (days), median (IQR)	1,376 (1,136–1,710)
**Complications**	
Mass effect	2 (12.5%)
Major ischemic stroke	1 (6.25%)
Foreshortening of stent	1 (6.25%)
**Follow-up mRS score**	
0–2	15 (93.75%)
3–5	0 (0%)
6	1 (6.25%)
Angiographic follow-up time (days), median (IQR)	246 (171–459)
**Follow-up aneurysm occlusion (OKM)**	
C	1 (6.67%)
D	14 (93.33%)
Mortality	1 (6.25%)

PED, pipeline embolization device; IQR, interquartile range; mRS, modified Rankin Scale; OKM, O'Kelly-Marotta grading scale.

### Case presentation 1

**Figure 1:**
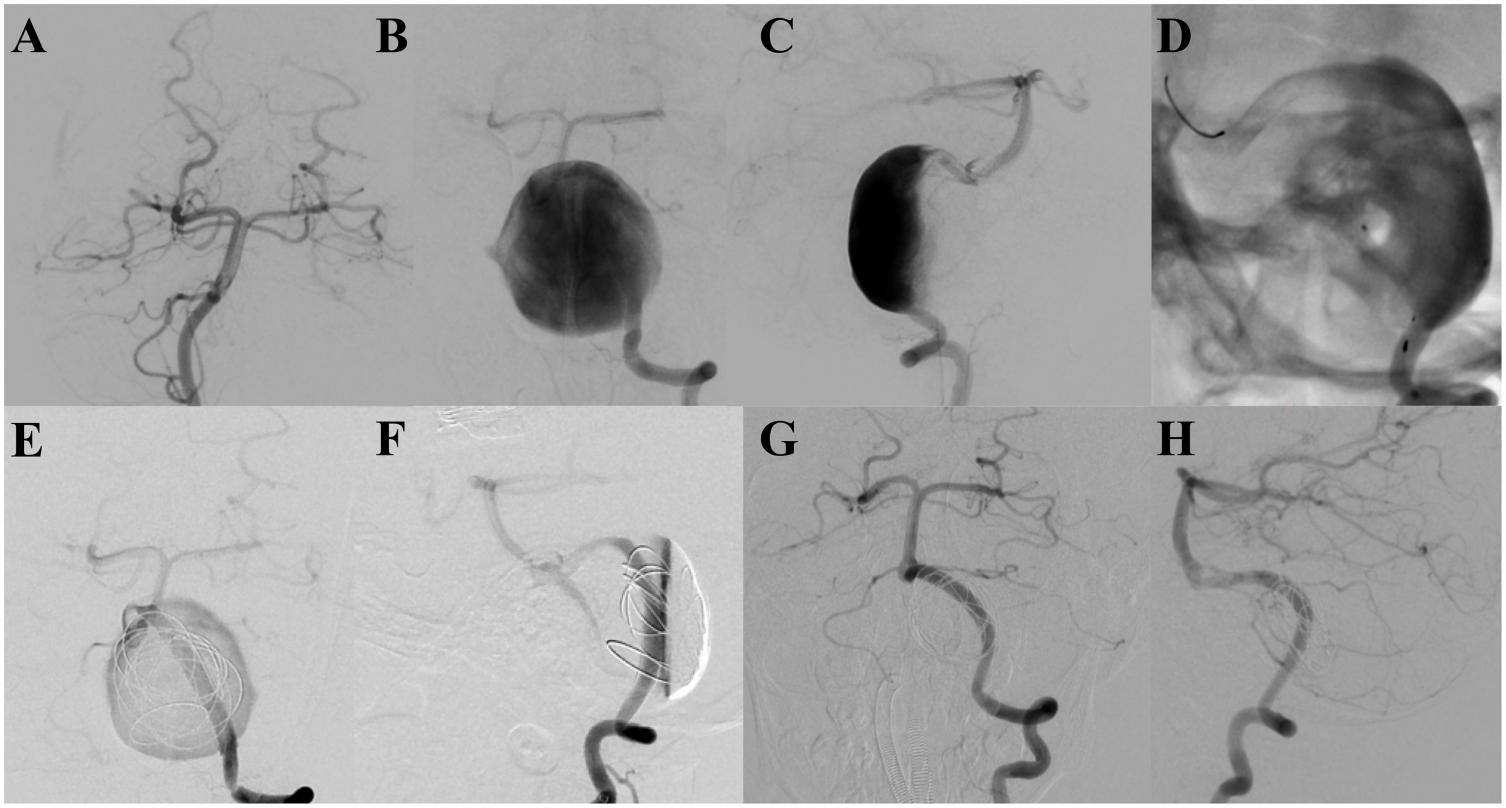
**(A–C)** Pre-operative digital subtraction angiography indicates a giant dissecting aneurysm located at the end of the left vertebral artery. **(D)** Three overlapping and successfully implanted pipeline embolization devices are shown. **(E, F)** Digital subtraction angiography analysis, showing the retention of contrast media in the aneurysm after the treatment. **(G, H)** Follow-up digital subtraction angiography, revealing the aneurysm to be completely occluded 9 months after the procedure.

A 4-year-old male patient presented with intermittent dizziness for 8 months, which had become worse in the last 1 month. Magnetic resonance angiography indicated a circular abnormal signal in front of the pons, and a left vertebral artery aneurysm was suspected. Furthermore, digital subtraction angiography (DSA) revealed a giant dissecting aneurysm located at the left vertebral artery with a maximum diameter of 39.5 mm. Three PEDs with adjunctive coils were successfully inserted to treat the aneurysms. The patient was kept under anesthesia for 48 h post-procedure; after waking, the patient was unable to swallow autonomously owing to mass effect, and a gastric tube was inserted. Ten days post-surgery, the patient developed dyspnea secondary to pneumonia and was intubated through a tracheal tube with ventilator-assisted breathing. Thirty days after the operation, tracheotomy was performed. The patient recovered 55 days after the procedure, without any neurological sequelae. Nine months later, follow-up DSA showed that the aneurysm was completely occluded, with a patient mRS score of zero (Figure 1).

### Case presentation 2

**Figure 2:**
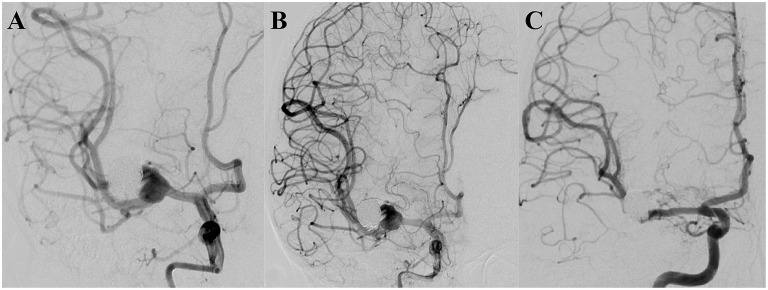
**(A)** Pre-operative digital subtraction angiography showing the aneurysm to be recurrent. **(B)** A successfully implanted single pipeline embolization device, and digital subtraction angiography analysis, revealing the retention of contrast media in the aneurysm. **(C)** Follow-up digital subtraction angiography, 3 months after the operation, indicating the aneurysm to be completely occluded.

A 16-year-old male patient was diagnosed with a dissecting aneurysm located at the right M1 segment of the middle cerebral artery and treated with interventional embolization with coils. Four months later, follow-up DSA revealed recurrence of the aneurysm, and subsequent clipping of the aneurysm was performed. After 15 months, follow-up DSA showed that the aneurysm recurred with a maximum diameter of 16.2 mm. After a comprehensive evaluation, a single PED without adjunctive coils was successfully used to treat the aneurysm. Three months later, follow-up DSA indicated that the aneurysm was completely occluded, and the mRS score was zero (Figure 2).

## Discussion

Pediatric nonsaccular intracranial aneurysms are rare but challenging lesions. These aneurysms typically manifest with fusiform or dissecting morphology, causing a range of neurological symptoms that can be life-threatening (1–3). In recent years, PEDs have gained popularity for the treatment of intracranial aneurysms. Previous studies have indicated that PEDs can be used to treat pediatric intracranial aneurysms (8–10, 12, 13). However, there is minimal evidence on PED safety and efficacy in pediatric nonsaccular aneurysm patients. In this study, we retrospectively examined 16 pediatric patients with 16 nonsaccular aneurysms treated with PEDs. The complication rate was found to be 25%. Finally, 93.75% of the patients achieved favorable outcomes and 93.33% achieved complete occlusion without any neurologic deficits. These findings suggest that PEDs are a safe and effective treatment method for pediatric nonsaccular aneurysms.

Mass effect is a complex complication of PED treatment for intracranial aneurysms. This complication often occurs in large or giant aneurysms, especially those in the posterior circulation, which are adjacent to important brainstem structures and cranial nerves (14, 15). PEDs can effectively reduce blood flow through the aneurysm and promote thrombus formation, allowing neointimal hyperplasia over their surface to reconstruct the parent artery and heal the aneurysm. The literature suggests that during the acute stage of intra-aneurysm thrombosis, aneurysm volume increases, leading to or exacerbating the mass effect. However, as time passes after PED implantation, the aneurysm shrinks in size, and the mass effect diminishes or disappears (16–19). The previous study indicates that usage of corticosteroids could reduce the occurrence of mass effect in adults, which might also be applicable in children (20). In this study, our findings were similar to those reported in previous studies. Two patients developed a mass effect after PED implantation; the aneurysms in both patients were large vertebrobasilar artery aneurysms adjacent to the brainstem and important cranial nerve nuclei. One patient died of respiratory and circulatory failure secondary to mass effect, at 3 days post-procedure. Another patient experienced dysphagia after recovering from anesthesia at 2 days post-procedure, and owing to the mass effect, and a gastric tube was inserted. The patient recovered 55 days after the procedure without any neurological complications. In clinical practice, we found that strict blood pressure control, careful nursing, and timely treatment of mass effect can prevent the occurrence of mass effects or reduce associated outcomes, including disability and death.

It is generally complicated and difficult to manage recurrent aneurysms after coiling, stenting, stent-assisted coiling, and surgical clipping. The application of PEDs in treating these aneurysms leads to favorable outcomes, with high complete occlusion rates and low complication rates (21–25). However, little evidence exists on the safety and effectiveness of PED treatment for recurrent pediatric aneurysms. This study included two pediatric patients with recurrent aneurysms. One patient had a recurrent aneurysm located at the C4 segment of the left internal carotid artery, which had previously undergone Willis covered stent placement. We successfully treated the aneurysm with a single PED implantation, and imaging follow-up 7 months later demonstrated complete aneurysm occlusion. Another patient had a recurrent aneurysm located at the M1 segment of the middle cerebral artery that had undergone two treatments previously: endovascular coiling and surgical clipping. The patient was treated with PED implantation without adjunctive coils, and follow-up imaging showed complete occlusion of the aneurysm 3 months after the operation. None of the patients had neurological complications. Our results suggest that PEDs are a potentially effective method for the treatment of recurrent aneurysms in pediatric patients.

Previous studies have confirmed that the use of specific doses of dual antiplatelet therapy in adults can reduce thromboembolic complications after PED placement (26–28). However, there are few studies on antiplatelet regimens after PED placement in pediatric patients. Therefore, the pediatric antiplatelet therapy remains controversial. A randomized controlled trial on heart disease in children aged 0–24 months demonstrated that administration of 0.2 mg/kg clopidogrel per day achieves a platelet inhibition level comparable to taking 75 mg per day in adults (29). Another comparative study suggested age-specific differences in agonist-simulated platelet reactivity in children compared to that in adults and highlighted the need for age-specific reference ranges for platelet function in children (30). These studies suggest differences between the use of antiplatelet drugs in children and adults. In a previous study on the use of flow diverters to treat pediatric cerebrovascular diseases, the authors administered different doses of aspirin and clopidogrel depending on whether the patients weighed more than 45 kg and achieved favorable clinical outcomes (31). In this study, the dosage of antiplatelet drugs was determined by considering the previous literature, the experience of neurointerventionists, and the specific situation of the patients. The included patients of our study were generally older, with the youngest patient being 4 years old and the rest being over 5 years old. To prevent thromboembolic complications, we administered an adult dose of aspirin and clopidogrel for patients older than 5 years. Considering the young age and low body weight, we halved both doses for the 4-year-old patients to prevent the occurrence of potential hemorrhage. In our study, a 10-year-old male patient experienced major ischemic stroke 2 months after the operation; the patient recovered 9 days after conservative treatment without any neurological complications. In summary, the dosage of using antiplatelet drugs in pediatric patients treated with flow diverters requires further investigation. Subsequent studies with larger case numbers are needed to explore the optimal antiplatelet therapy regimen.

## Limitation

This study has several limitations. First, this was a retrospective study with a relatively small sample size and a short follow-up duration. Due to the small sample size, we could not explore factors influencing the complications and embolization outcomes. Additionally, as the youngest patient in our study was 4 years old, we were unable to determine the safety and effectiveness of PED treatment for these aneurysms in children younger than 4 years.

## Conclusions

In this cohort, we achieved a high rate of complete aneurysm occlusion and favorable clinical follow-up outcomes. These findings suggest that the use of PED to treat pediatric nonsaccular aneurysms is safe and effective.

## Data availability statement

The original contributions presented in the study are included in the article/supplementary material, further inquiries can be directed to the corresponding authors.

## Ethics statement

Ethical review and approval was not required for the study on human participants in accordance with the local legislation and institutional requirements. Written informed consent to participate in this study was provided by the participants' legal guardian/next of kin.

## Author contributions

JH and FL collected and analyzed the data and drafted and revised the paper. YuxZ monitored data collection and analyzed the data. YupZ and SL revised the paper and gave final approval of the version to be submitted. HZ, YC, and CM monitored data collection. LL revised the paper critically and gave final approval of the version to be submitted. CJ and ZJ concepted and designed the article, revised the paper critically, and gave final approval of the version to be submitted. All authors contributed to the article and approved the submitted version.
